# Effects of Strychnine on the Stomach

**Published:** 1891-09

**Authors:** 


### Effects of Strychnine on the Stomach.

The effect of nitrate of strychnine on the functional activity of the stomach has been recently made the subject of a careful research by Dr. Gamper, of St. Petersburg, who employed for the purpose of his experiments four healthy young hospital assistants. He found that strychnine increased the amount of gastric juice secreted, the general acidity, and the quantity of free acid in the secretion. It also hastened the absorption from the stomach, and strengthened the mechanical movements. Its effect, too, continued for some time after its administrtion had been stopped. Like many other Russian observers Dr. Gamper seems to have been highly impressed by the value of strychnine in chronic alcoholism, declaring that it is the most effective of all drugs in such cases. The thesis contains a long list of references to the literature of stomach affections, published in six or seven languages during the last ten years.

Important Improvement in Microscope Lenses.—It is stated that an immense improvement has recently been effected in the manufacture of glass for optical instruments by means of the addition to the ordinary materials of phosphorus and chlorine, which in some as yet unexplained way cause the glass to be very much more transparent, and enable it to receive a much higher degree of polish than any optical glass hitherto manufactured. Thus microscopes can be made which will render objects of the diameter of only the one-eighth millionth of a millimetre visible, whereas with the best instruments now in use the diameter of the smallest object that can be seen in one-sixteenth thousandth of a millimetre.

### Physiology in the Schools.

The following question and answer record what actually happened in a "district school" in the State of Pennsylvania, in a junior class in physiology:

Teacher.—What teeth come last?

Pupil.—False teeth.

